# Morphological and molecular data on tadpoles of the westernmost Himalayan spiny frog *Allopaa hazarensis* (Dubois & Khan, 1979)

**DOI:** 10.3897/zookeys.1049.66645

**Published:** 2021-07-20

**Authors:** Sylvia Hofmann, Rafaqat Masroor, Daniel Jablonski

**Affiliations:** 1 Centre of Taxonomy and Evolutionary Research, Zoological Research Museum Alexander Koenig, Adenauerallee 160, D-53113 Bonn, Germany Zoological Research Museum Alexander Koenig Bonn Germany; 2 Helmholtz-Centre for Environmental Research – UFZ, Department of Conservation Biology, Permoserstrasse 15, D-04318 Leipzig, Germany Helmholtz-Centre for Environmental Research Leipzig Germany; 3 Zoological Sciences Division, Pakistan Museum of Natural History, Garden Avenue, Islamabad 44000, Pakistan Pakistan Museum of Natural History Islamabad Pakistan; 4 Department of Zoology, Comenius University in Bratislava, Ilkovičova 6, Mlynská dolina, 842 15 Bratislava, Slovakia Comenius University Bratislava Slovakia

**Keywords:** *
Chaparana
*, Dicroglossidae, DNA barcoding, larva, *
Paa
*, Pakistan, species identification

## Abstract

Little is known about the life history, ecology, and distribution of the genus *Allopaa* (Dicroglossidae) and far less recent data are available about the larvae of this taxon. Here, we provide data on the larval stage of *Allopaa hazarensis* (Dubois & Khan, 1979) from northern Pakistan based on the examination of three tadpoles. Specimens were obtained from two sites in Buner, Khyber Pakhtunkhwa province, Pakistan. Morphological and genetic analysis (mtDNA and nDNA) confirmed the identity of the tadpoles as *A. hazarensis*. Tadpole characterizations were illustrated by detailed imagery. Basic measurements and details on oral apparatus provide relevant taxonomic characteristics to distinguish the tadpoles of this species from other spiny frogs. The illustration and description of the tadpole of *A. hazarensis* should facilitate the identification of this species in the field.

## Introduction

The Hazara (Torrent) Frog, *Allopaa hazarensis* (Dubois & Khan, 1979), belongs to the tribe Paini (Dicroglossidae), which are found across the Himalayan mountain arc from northern Afghanistan, Pakistan, and northern India, through Nepal, Sikkim, and Bhutan, and in the valleys of southern and eastern Tibet, eastwards to eastern China, and southwards to the mountains of Indochina (Myanmar, Thailand, Laos, northern Vietnam; Frost 2021). They live mostly in boulder-rich streams (Dubois 1975) or clear pools with flowing water (Khan et al. 2008; Ahmed et al. 2020; own observation). Males are characterized by black, keratinous spines, scattered on the fingers, arms, breast, or belly (Ohler and Dubois 2006). The Paini tribe is currently composed of the genus *Quasipaa* Dubois, 1992 (11 species), *Nanorana* Günther, 1896 (around 30 species), *Allopaa* Ohler & Dubois, 2006 (possibly two species), and the monotypic genus *Chrysopaa* Ohler & Dubois, 2006, with *C. sternosignata* (Murray, 1885). The latter two genera represent the most western dicroglossid frogs that occur in the Himalayan-Tibetan orogenic belt (HTO). Recently, the phylogenetic placement of *Allopaa* from Kashmir Himalaya and *Chrysopaa* from the Hindu Kush has been addressed for the first time (Hofmann et al. 2021). This study indicates no close taxonomic relations between the two genera and their geographical neighbouring spiny frogs and suggests a trans-Tibet dispersal during the late Oligocene (*Chrysopaa*) and early Miocene (*Allopaa*) from the eastern margin of the HTO.

*Allopaa hazarensis* was described from near Datta (~34.30°N, 73.26°E), northern Pakistan (Manshera District, Hazera Division, about 1,200 m a.s.l.) and is known to occur in Khyber Pakhtunkhwa Province, Pakistan, and in adjacent Kashmir, India (see Frost 2021). It has been also reported from Taluka Kotri and Thano Bula Khan of District Jamshoro (~25.35°N, 68.27°E; ~25.36°N, 67.84°E), southern Pakistan, but no photographs and morphological or molecular data were provided (Shaikh et al. 2015). Little is known about the life history, ecology, and distribution of *Allopaa*, and far less recent data are available about the larvae of this taxon. *Allopaa hazarensis* can be found in pools of clear water in small creeks or torrents running in deep gorges. Breeding starts probably in June with the first summer rain. No acoustic data of the mating call exist, and it is still unknown whether tadpoles may develop from eggs deposited the preceding year or if larvae can over-winter in water (Dubois and Khan 1979). Tadpoles of *A. hazarensis* were morphologically described in the original species description (Dubois and Khan 1979). Further details on the oropharyngeal morphology of the larva in relation to feeding mechanisms in supposedly torrenticole habitats were provided by Khan and Malik (1987).

In the present study, we provide detailed photographs and a brief description of *A. hazarensis* tadpoles from northern Pakistan. We used mitochondrial and nuclear DNA sequence data to validate the identity of our specimens by assigning them to existing *Allopaa* sequences. These recent data may support future research on this taxon in the Kashmir Himalaya and adjacent regions.

## Methods

### Sampling, illustrations, and character assessment

**Figure 1.**
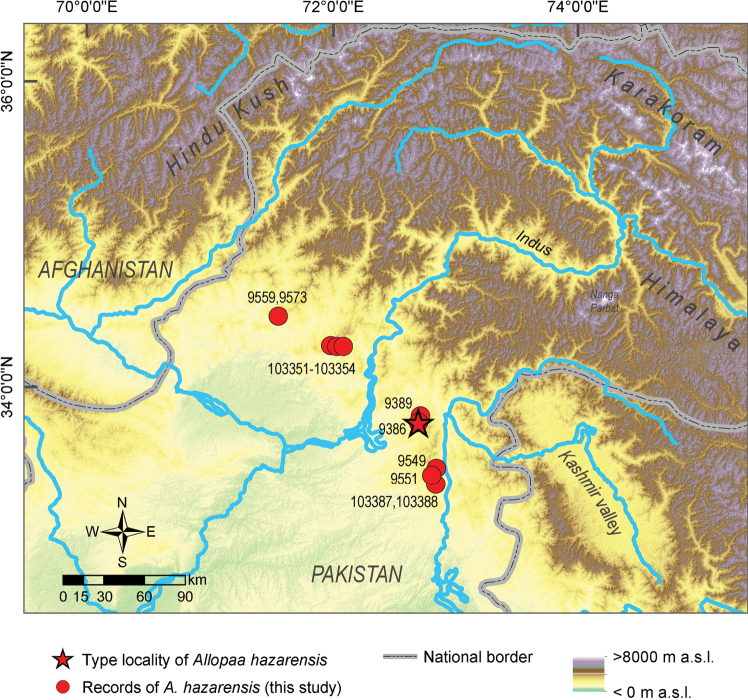
Map showing the locations of the *Allopaa hazarensis* specimens reported herein and further known records (for details, see Suppl. material 1: Table S1). The type locality of *A. hazarensis* is indicated by a star.

**Figure 2.**
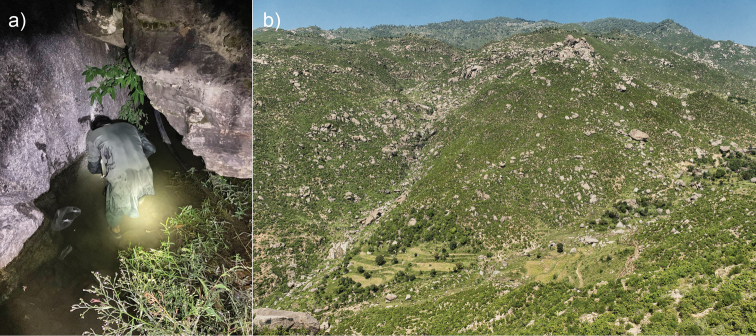
Typical habitat of *Allopaa hazarensis* from Buner, Khyber Pakhtunkhwa province, Pakistan (34.64°N, 72.47°E, 935 m a.s.l.) **a** collection site of one of the tadpoles (ZFMK 103351) **b** overview of the collection site. Photo credit: Daniel Jablonski.

Two larvae (ZFMK 103353, ZFMK 103354) were collected in September 2020 during night time in Buner, Khyber Pakhtunkhwa province, Pakistan (34.66°N, 72.50°E, 1,520 m a.s.l.; Fig. 1, Suppl. material 1: Table S1). An additional tadpole (ZFMK 103351) was collected at lower elevation near Qadir Nagar River, Buner, Khyber Pakhtunkhwa province, Pakistan (34.64°N, 72.47°E, 935 m a.s.l.). The larvae were observed in clear water pools of a boulder-rich torrents (Fig. 2).

Sampling was conducted according to the regulations for the protection of terrestrial wild animals under the permits of the Pakistan Museum of Natural History, Islamabad, Pakistan [no. PMNH/EST-1(89)/05]. A small piece of the tail was taken from two of the tadpoles (ZFMK 103354, ZFMK 103351), transferred into absolute ethanol, and stored at −20 °C. All investigated specimens are deposited in the Zoological Research Museum Alexander Koenig, Bonn, Germany.

Tadpoles were staged according to Gosner (1960), preserved in 70% ethanol, and morphologically described under a stereomicroscope. Photos of entire tadpoles were taken at the lab with a Nikon D750 digital camera, a 105 mm macro lens, and a 2.0× teleconverter for detail shots. Morphometric measurements were taken with digital calipers (accuracy ± 0.1 mm). Tadpole terminology follows Altig and McDiarmid (1999); the following measurements were taken: TL (total length), BL (body length), TAL (tail length), TMH (tail muscle height at tail base), TMW (tail muscle width at tail base), IOD (interorbital distance), IND (inter-narial distance), EN (eye-nostril distance), ODW (oral disc width). All measurements are provided in Suppl. material 1: Table S2. Characteristics of the oral disc were described according to the system suggested by Altig (1970). The formula of keratodonts (= labial tooth rows) is abbreviated LTRF and is presented according to Altig and McDiarmid (1999), with the anterior (A-) and posterior (P-) rows indicating gaps in brackets and a backslash separating the upper and lower jaw sheaths (Schulze et al. 2015).

### DNA extraction, sequence alignment and phylogenetic reconstruction

Genomic DNA was extracted from tissues using the DNeasy Blood & Tissue Kit (Qiagen, Venlo, The Netherlands) following the manufacturer’s protocol. Approximately 546 bp of the 16S ribosomal RNA (rRNA), 499 bp of the Cytochrome c oxidase I (COI), and a fragment of 1,207 bp of the Recombination activating gene 1 (Rag1) gene were amplified via the polymerase chain reaction (PCR) using primers and PCR conditions as previously described (Hofmann et al. 2019). PCR products were purified using the mi-PCR Purification Kit (Metabion, Planegg, Germany) and sequenced in both directions by Macrogen (Amsterdam, Netherlands; http://www.macrogen.com).

We aligned the new sequences (accession numbers 16S: MW723172–MW723173, COI: MW723177–MW723178, Rag1: MW728951–MW728952) to data available from our previous studies (Hofmann et al. 2019, 2021) by eye; for accession numbers and detailed information of these previous data see supplementary table in Hofmann et al. (2021). The 16S sequences were aligned based on secondary structures. Alignment based on amino acids produced similar results, since no ambiguities, such as deletions, insertions, or stop codons, were found. The final concatenated rRNA + mtDNA + nuDNA sequence dataset consisted of 183 taxa and contained 2,317 alignment positions of which 494 were phylogenetically informative. We inferred a maximum-likelihood (ML) tree using RAxML v. 8.2.12 (Stamatakis 2014). The dataset was partitioned a priori by gene and codon fragments, and PartitionFinder v. 1.1.1 (Lanfear et al. 2012) was applied to optimize partitions using linked branch lengths, the corrected Aikaike Information Criterion (AICc), the greedy search algorithm, and the substitution models implemented in RAxML. We ran RAxML with the GTRGAMMA model and 1,000 bootstrap replicates on CIPRES (Cyberinfrastructure for Phylogenetic Research) (Miller et al. 2010).

## Results

### Phylogenetic assignment

We confirmed the identity of the two tadpoles as *Allopaa hazarensis*. Our tadpole sequences nested in the clade of *A. hazarensis* that includes sequences from the type locality; the placement within this clade was highly supported (Suppl. material 1: Fig. S1).

### Tadpole characterization

**Figure 3.**
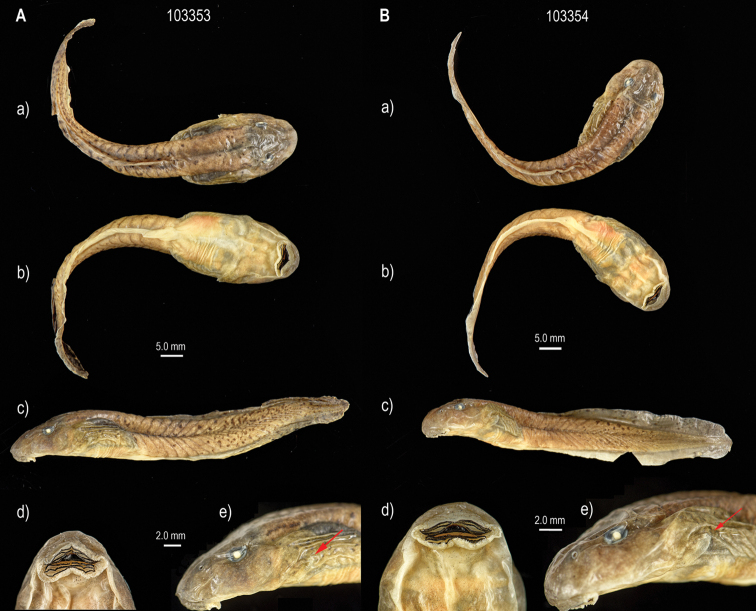
Tadpoles (Gosner stage 26) of *Allopaa hazarensis* from Buner, Khyber Pakhtunkhwa province, Pakistan, 1520 m **a–c** dorsal, ventral, lateral views **d** mouthpart **e** spiracle of preserved specimens (**A** ZFMK 103353 **B** ZFMK 103354).

**Figure 4.**
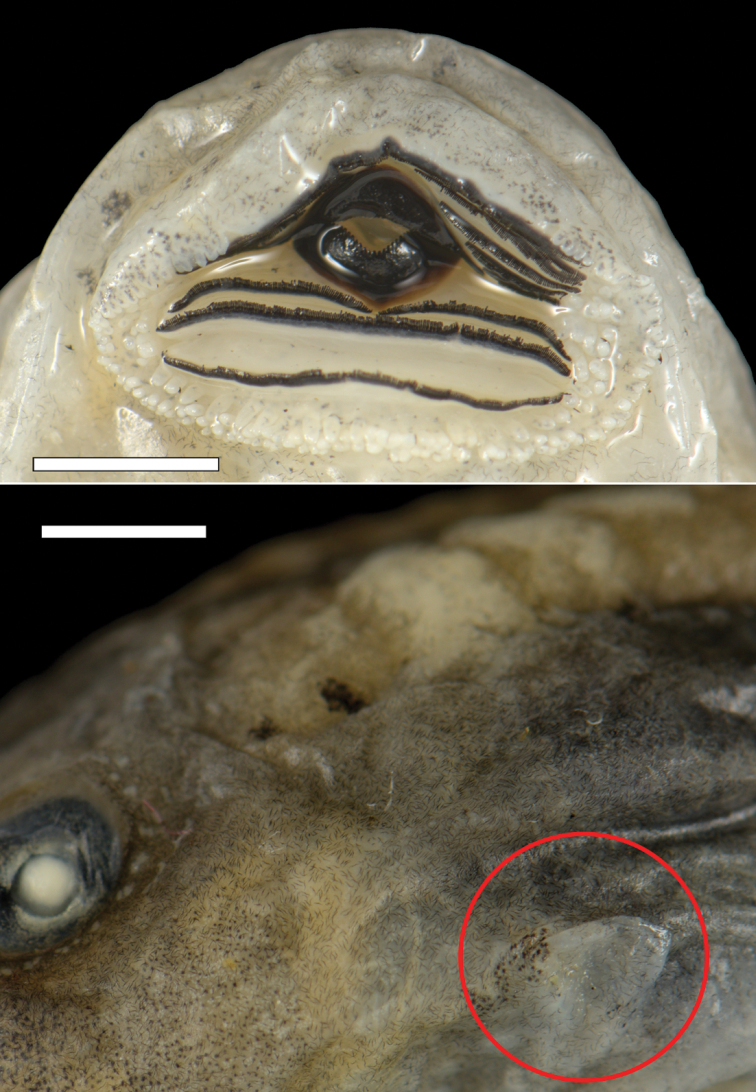
Close up of the oral disc and spiracle of a preserved tadpole of *Allopaa hazarensis* larva (ZFMK 103351; Gosner stage 26). Scale bars: 2 mm.

Tadpoles identified as *A. hazarensis* have been described by Khan and Dubois (1979) and Khan and Malik (1987). These morphological descriptions agree with the specimens studied herein in terms of body shape, coloration, caudal muscle, and oral disc, but differ in some basic measurements, details on oral apparatus, and Gosner stage. Thus, the following brief description is based on three specimens (ZFMK 103351, ZFMK 103353, ZFMK 103354) at Gosner stages 26 from Buner, Pakistan: total length 61.6, 76.5, and 73.2 mm, body length 19.8, 25.3, and 22.8 mm, respectively (for details see Suppl. material 1: Table S2). Large body, oblong-ovoid in dorsal and ventral views, compressed in lateral view; snout semicircular rounded in dorsal and ventral views, and slightly sloped in lateral view (Fig. 3a–c; Suppl. material 1: Fig. S2). Eyes and nostrils small, located and directed dorsolaterally. Oral disc large, located and directed ventrally to anteroventrally (Fig. 3d). Marginal papillae uniseriate with a wide median gap on upper labium, biseriate anterolaterally to laterally and on lower labium. Submarginal papillae present and cumulated laterally, in the wrinkle between labia (Figs 3d, e, 4). Jaw sheaths robust and finely serrated, the upper sheath slightly arc-shaped, the lower V-shaped; LKRF 8(2–8)/3(1), or 7(2–7)/3(1) (see Suppl. material 1: Table S1). Spiracle sinistral, opening posterodorsally (Fig. 4).

## Discussion

Here we report on larvae of the dicroglossid frog *Allopaa hazarensis*. Our work is based on morphological and DNA data of *A. hazarensis* tadpoles and previously compiled molecular data sets (Hofmann et al. 2019, 2021). The study provides the first photographs of *A. hazarensis* larvae, supporting morphological data, and additional distribution records of the species.

Our new data mainly agree with previous descriptions (Dubois and Khan 1979; Khan and Malik 1987) in characterizing *A. hazarensis* tadpoles as typical ranoid type. These larvae have a large body (19.8–25.3 mm) with a strong muscular tail. In the original description, body lengths of five tadpoles ranged between 13.7 and 25.3 mm (stages 25–42); Khan and Dubois (1987) reported 25 mm for 11 tadpoles at stage 40. The mouth is located almost ventrally but close to tip of snout. The oral disc is prominent, bordered by marginal papillae with a larger gap on the upper lip, with multiple submarginal papillae at the libs’ commissure, and the serrated jaws are robust. The number of keratodonts varies between seven and eight in the anterior part of the mouth with A_2_–A_n_ being discontinous rows, while three labial tooth rows are present in the posterior part (P_1_ discontinous), which is consistent with the description of Khan and Malik (1987). However, in the original description, a tadpole at stage 25 with only six tooth rows on the upper labia has been also reported (Dubois and Khan 1979).

Based on the original description, *A. hazarensis* has been assumed to be most closely related to *Nanorana minica* (Dubois, 1995), which has been reported to occur in Indian Uttar Pradesh and Himachal Pradesh, and in western Nepal (Frost 2021). The tadpoles of the two species have been denoted to be “quite similar” (Dubois and Khan 1979). However, phylogenetically, *Allopaa* does not cluster together with geographically neighboring species of the subgenus Paa from India and Nepal, but with the subgenus Chaparana from montane regions of the southeastern margin of the Tibetan Plateau and mountains of NE China (Suppl. material 1: Fig. S2). This corroborates the strong morphological differences between adult *A. hazarensis* and *N. minica* and the lack of the typical characteristics of the subgenus Paa in *A. hazarensis*, namely, the very prominent secondary sex characters in males, and the large size and the low number of the eggs, which have been considered to represent adaptive features related to breeding in swiftly running torrents (Dubois and Khan 1979). All specimens mentioned in the present study were observed in pools (1–4 m in diameter) of small to medium-sized streams under warm-temperate conditions in the foothill to lower montane zone (comparable to those of its sister group *Chaparana*; Ohler et al. 2000; Che et al. 2010). Thus, given the habitats in which we found *A. hazarensis*, we agree with the assumption that, in contrast to *Paa*, this taxon is not truly a torrent species but occupies clear water pools of boulder-rich creeks (Dubois and Khan 1979). We also suspect that larvae of *A. hazarensis* can overwinter in shallow standing or flowing permanent waters, as we found tadpoles at early Gosner stages repeatedly in September, making it unlikely that they will metamorphose before the winter.

Our tadpoles differ significantly from those of *Nanorana vicina* (Stoliczka, 1872), which is endemic to uplands in northern Pakistan and India. Compared to *A. hazarensis*, the number of tooth rows on the upper labia of the *N. vicina* tadpoles is fewer (5 *vs.* 7 or 8; no difference on lower labia), and the submarginal papillae are not cumulated laterally (Gill et al. 2020). Furthermore, except for *N. taihangnica*, the keratodont row formula for the upper labia differs between *A. hazarensis* and *Chaparana* (*N. aenea*, *N. quadranus*, *N. unculuanus*, *N. yunnanensis*), several *Paa* species (*N. chayuensis*, *N. conaensis*, *N. maculosa*, *N. medogensis*, and all *Nanorana* species (*N. parkeri*, *N. pleskei*, *N. ventripunctata*; Fei et al. 2012; Chuaynkern et al. 2018). In contrast, tadpoles of the geographically neighboring *Chrysopaa sternosignata* share the same number of keratodonts on the upper and lower labia as *A. hazarensis* (Ohler and Dubois 2006).

## Conclusions

The illustration and description of the tadpole of *A. hazarensis* should facilitate the identification of this species, for example, during tadpole surveys in Pakistan. As the tadpoles of this species need several weeks to even months to complete development, they can be often more easily detected than their adult conspecifics, both at night and during the day. The sparse knowledge about the genus *Allopaa*, and particularly of their larval stages, requires more research to utilize the valuable tadpole data, especially for monitoring and conservation efforts.

## Supplementary materials

Supplementary material 1 Tables S1, S2, Figures S1, S2 Data type occurences, morphological, and phylogenetic Explanation note List of *Allopaa hazarensis* specimens used in the present study; Measurements (in mm) and counts of voucher specimens (V-ID) of tadpole series of *Allopaa hazarensis*; Maximum-likelihood tree based on concatenated mtDNA and nuDNA sequence data; Tadpole (103351; Gosner stage 26) of *Allopaa hazarensis* from Buner, Pakistan. https://binary.pensoft.net/file/568604This dataset is made available under the Open Database License (http://opendatacommons.org/licenses/odbl/1.0/). The Open Database License (ODbL) is a license agreement intended to allow users to freely share, modify, and use this Dataset while maintaining this same freedom for others, provided that the original source and author(s) are credited.Sylvia Hofmann, Rafaqat Masroor, Daniel Jablonski
