# Supplementary material for: Morphological and molecular data on tadpoles of the westernmost Himalayan spiny frog Allopaa hazarensis (Dubois & Khan, 1979)
Source: Zookeys. 2021 Jul 20;1049:67–77. doi: 10.3897/zookeys.1049.66645 (PMC8316248; doi:10.3897/zookeys.1049.66645)

Supplementary information

Morphological and molecular data on tadpoles of the westernmost Himalayan spiny frog *Allopaa hazarensis* (Dubois and Khan, 1979)

Sylvia Hofmann, Rafaqat Masroor, Daniel Jablonski

Table S1. List of *Allopaa hazarensis* specimens used in the present study (indicated bold) and previous work, as shown in Fig. 1, including sample ID or voucher number, and sample localities. The holotype is marked with an asterisk. Coordinates are given in decimal degrees. ZFMK = Zoological Research Museum Alexander Koenig, Bonn, Germany

ID/No	Locality	N	E	Altitude
**ZFMK 103351**	Qadir Nagar, Buner, Khyber, Pakistan	34.6401	72.4716	935
ZFMK 103352	Buner, Khyber, Pakistan	34.6564	72.4961	1520
**ZFMK 103353**	Buner, Khyber, Pakistan	34.6564	72.4961	1520
**ZFMK 103354**	Buner, Khyber, Pakistan	34.6564	72.4961	1520
ZFMK 103387	near Murree, Pakistan	33.8596	73.4701	1638
ZFMK 103388	near Murree, Pakistan	33.8596	73.4701	1638
9386	Datta, Manshera, Pakistan	34.2950	73.2580	1300
9389	Datta, Manshera, Pakistan	34.2950	73.2580	1300
9549	Aliot, Pakistan	33.9510	73.4680	1511
9551	Margi, Pakistan	33.9400	73.4650	1618
9559	Laram Qilla, Lower Dir, Pakistan	34.7850	71.9860	1436
9573	Laram Qilla, Lower Dir, Pakistan	34.7850	71.9860	1436
1978.3056*	near Datta, Manshera, Hazera, Pakistan	34.2500	73.2500	1200

Table S2. Measurements (in mm) and counts of voucher specimens (V-ID) of tadpole series of *Allopaa hazarensis* deposited at the Zoological Research Museum Alexander Koenig, Bonn, Germany. TL: total length; BL: body length; TAL: tail length; TMW: tail muscle width at tail base; IOD: interorbital distance; TMH: tail muscle height at tail base; IND: internarial distance; EN: eye-nostril distance; OWD: oral disc width.

V-ID	TL	BL	TAL	TMH	TMW	IOD	IND	EN	ODW	LTRF
103351	61.6	19.8	41.8	7.0	5.9	5.6	3.4	4.0	6.3	7(2-7)/3(1)
103353	76.5	25.3	51.2	8.1	7.6	8.2	4.3	5.0	7.9	8(2-8)/3(1)
105554	73.2	22.8	50.4	6.8	7.0	7.4	4.5	4.7	8.2	8(2-8)/3(1,3)

Figure S1. Maximum-likelihood tree based on concatenated mtDNA and nuDNA sequence data. Branch nodes supported by bootstrap values >75 are marked with a small rectangle. In the *Allopaa* clade species name is followed by specimen ID in brackets; IDs of the two tadpoles deposited at the Zoological Research Museum Alexander Koenig, Bonn, Germany and described herein are in bold.

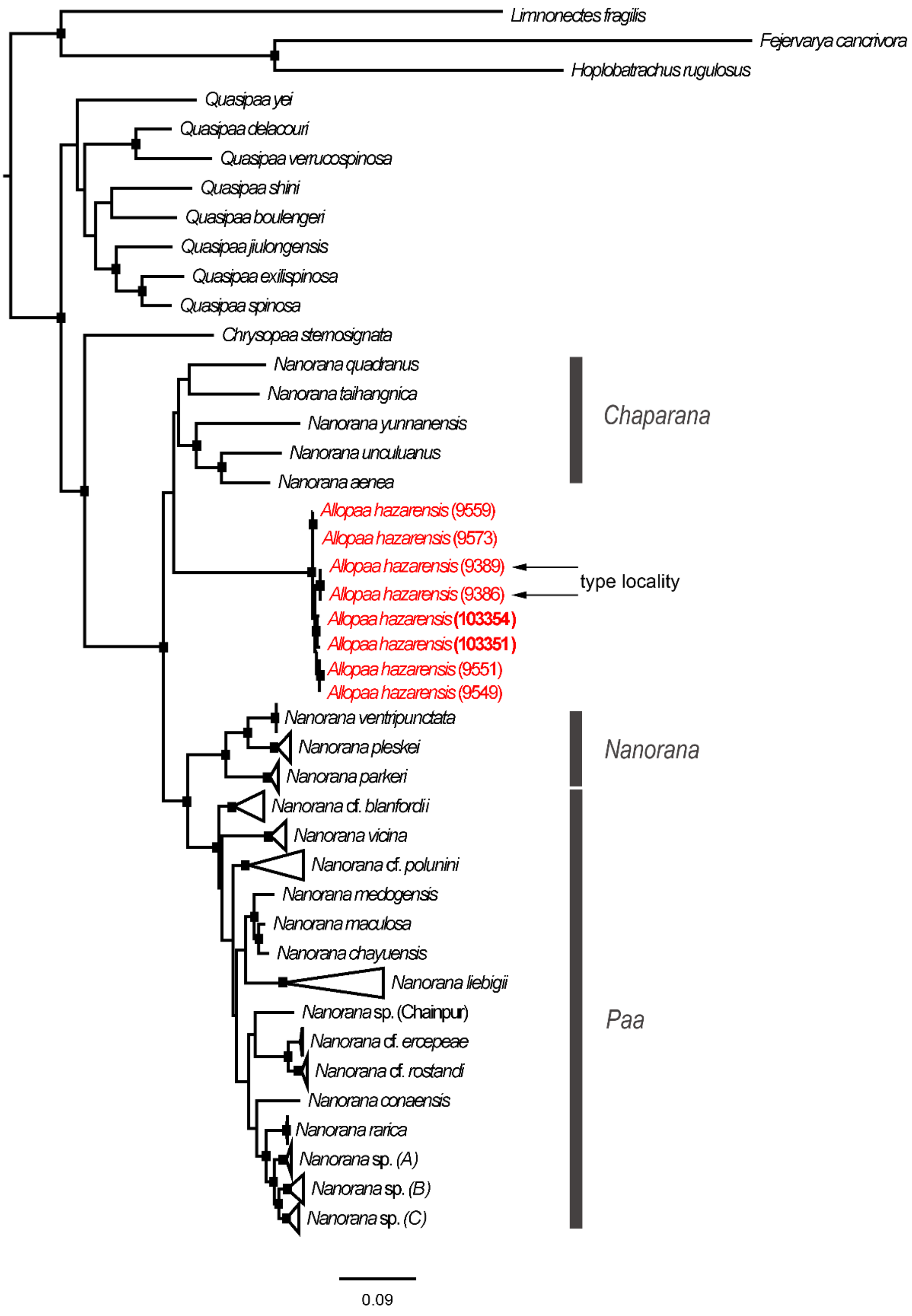

Figure S2. Tadpole (ZFMK 103351; Gosner stage 26) of *Allopaa hazarensis* from Buner, Khyber, Pakistan, 935 m; dorsal, lateral, and ventral view.

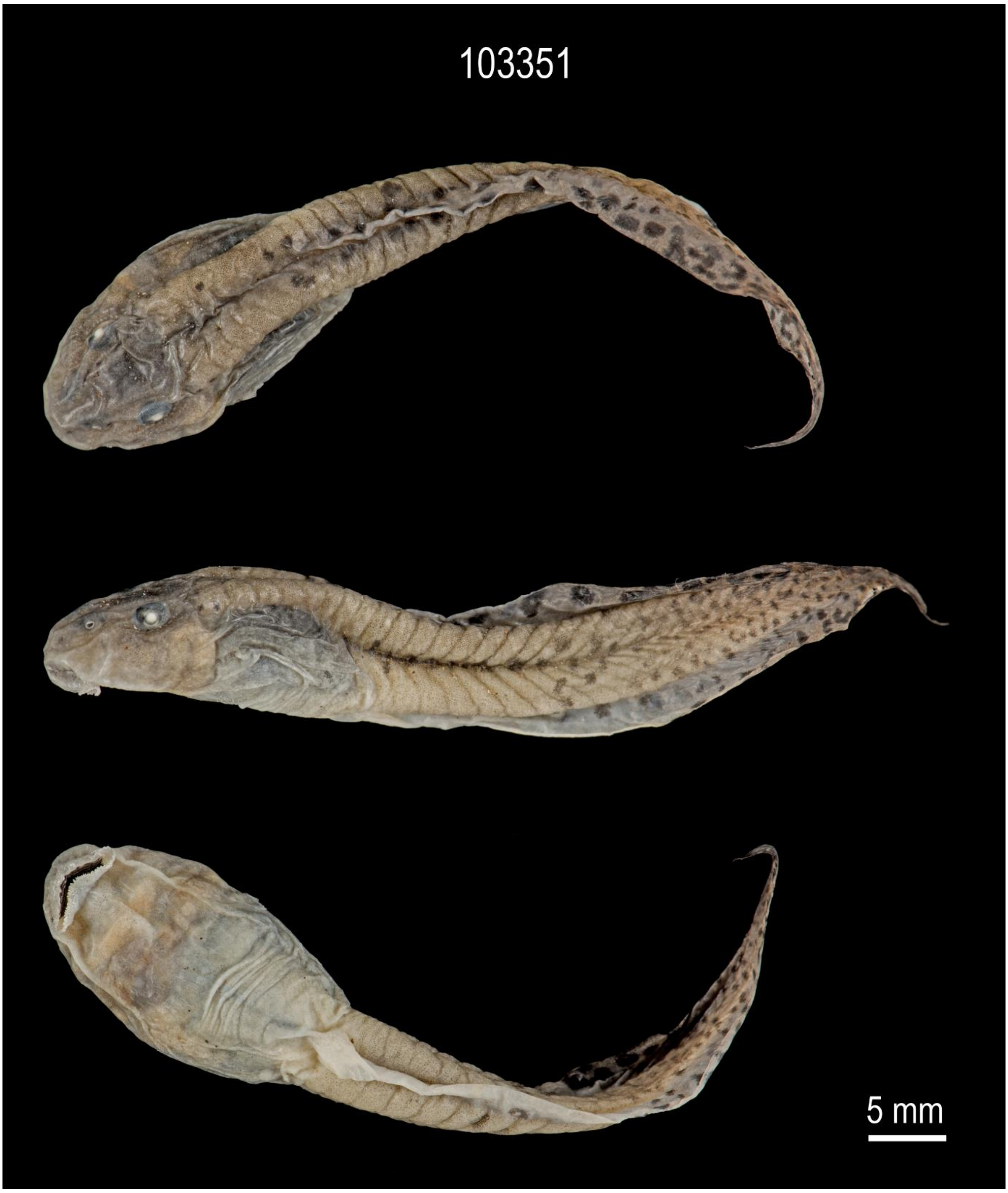

Supplement: Supplementary material 1 — Tables S1, S2, Figures S1, S2 [file zookeys-1049-067-s001.pdf]
